# Supplementary material for: External drivers of BOLD signal’s non-stationarity
Source: PLoS One. 2022 Sep 19;17(9):e0257580. doi: 10.1371/journal.pone.0257580 (PMC9484685; doi:10.1371/journal.pone.0257580)
Supplement: S1 File — (PDF) [file pone.0257580.s001.pdf]

Supporting Information (SI) for

"External drivers of BOLD signal's non-stationarity"

by Arian Ashourvan[1,9], Sérgio Pequito[2], Jason Z. Kim[3], Maxwell Bertolero[3], Danielle S. Bassett[3,4,5,6,7,8], and Brian Litt[3,4,5]

[1]Center for Neural Engineering, Department of Engineering Science and Mechanics, The Pennsylvania State University, University Park, PA, 16802 USA
[2]Delft Center for Systems and Control, Delft University of Technology, Netherlands
[3]Department of Bioengineering, University of Pennsylvania, Philadelphia, PA, 19104 USA
[4]Penn Center for Neuroengineering and Therapeutics, University of Pennsylvania, Philadelphia, PA, 19104 USA
[5]Department of Neurology, Hospital of the University of Pennsylvania, Philadelphia, PA, 19104 USA
[6]Department of Psychiatry, Perelman School of Medicine, University of Pennsylvania, Philadelphia, PA, 19104 USA
[7]Department of Electrical & Systems Engineering, School of Engineering and Applied Science, University of Pennsylvania, Philadelphia, PA, 19104 USA
[8]Department of Physics & Astronomy, College of Arts and Sciences, University of Pennsylvania, Philadelphia, PA, 19104 USA
[9]Correspondence to: Arian Ashourvan, The Pennsylvania State University, W311 Millennium Science Complex, Pollock Road, University Park, PA 16802-2131, Email: aba5983@psu.edu

Contents

SI1. Joint-estimation algorithm **3**

SI2. LTI system's output simulation **5**

SI3. The dimensionality of external inputs **6**

SI4. Determining the optimal number of eigenvector clusters **8**

SI5. The sparsity of system matrix and external inputs **11**

SI6. Effect of estimation window's length on input's accuracy **13**

SI7. Principal component analysis of LTI model's residuals **14**

Supplementary Figures **15**

References **36**

SI1. Joint-estimation algorithm.

In what follows, we seek to determine the dynamics and input matrices, as well as the input sequence of the following dynamical system

$$x[k+1] = Ax[k] + Bu[k], \tag{1}$$

where $x[k] \in \mathbb{R}^n$ denotes the state, $u[k] \in \mathbb{R}^p$ denotes the input, $A \in \mathbb{R}^{n\times n}$ the dynamics matrix, and $B \in \mathbb{R}^{n\times p}$ the input matrix. Specifically, by considering a sequence of data $\{z[k]\}_{k=0}^{N-1}$, we seek to find an approximation of the parameters $(A, B, \{u[k]\}_{k=0}^{N-1})$ denoted by $(\tilde{A}, \tilde{B}, \{\tilde{u}[k]\}_{k=0}^{N-1})$, respectively. Next, notice that given the dynamical system (1), we obtain

$$z[k] = Az[k-1] + Bu[k-1] + \varepsilon_k, \tag{2}$$

where the error can be captured by $\varepsilon_k \sim \mathcal{N}(0, \Sigma)$. Subsequently, in a least-squares minimization sense, we seek to solve the following minimization problem

$$\min_{(\tilde{A},\tilde{B},\{\tilde{u}[k]\}_{k=0}^{N-1})} \sum_{k=1}^{N} \|z[k] - \left(\tilde{A}z[k-1] + \tilde{B}\tilde{u}[k-1]\right)\|_2^2. \tag{3}$$

Additionally, since both the input matrix and sequence of inputs are unknown, the problem is not well posed, which force us to consider possible feasibility constraints (e.g., $\|\tilde{u}[k]\| \leq 1$), and the objective should be changed to account for a regularization term (e.g., the 1-norm) that penalize the number of non-zero entries, which lead to the following problem:

$$\min_{(\tilde{A},\tilde{B},\{\tilde{u}[k]\}_{k=0}^{N-1})} \sum_{k=1}^{N} \|z[k] - \left(\tilde{A}z[k-1] + \tilde{B}\tilde{u}[k-1]\right)\|_2^2 + \lambda\|\tilde{u}[k-1]\|_1^2 + \lambda\|\tilde{B}\|_1^2, \tag{4}$$

where $\lambda \in \mathbb{R}_0^+$ is the regularization term that weights the tradeoffs between the approximation error and the number of nonzero entries. To determine the unknown parameters $(\tilde{A}, \tilde{B}, \{\tilde{u}[k]\}_{k=0}^{N-1})$ that are the solution to the above problem, we will consider an algorithm that borrows ideas from the Expectation-Maximization algorithm[1], which details can be found in[2;3].

Briefly, we seek to determine a converging sequence of unknown parameters $(\tilde{B}^{(l)}, \{\tilde{u}^{(l)}[k]\}_{k=0}^{N-1})$, which initial parameters (i.e., with $l = 0$) are set as follows: (i) $\tilde{A}$ is the solution to (3) with input matrix and inputs set to zero; and (ii) $\tilde{B}^{(0)}$ is initialized at random which entries are obtained from standard Gaussians. Next, for a predefined value of λ in (4), we find a solution to it by proceeding sequentially as follows (for each iteration $l = 1, 2, \ldots$): (i) determine $\{\tilde{u}^{(l)}[k]\}_{k=0}^{N-1}$, given the values $(\tilde{A}, \tilde{B}^{(l-1)})$

$$\min_{\{\tilde{u}^{(l)}[k]\}_{k=0}^{N-1}} \sum_{k=1}^{N} \|z[k] - \Big(\tilde{A}z[k-1] + \tilde{B}^{(l-1)}\tilde{u}^{(l)}[k-1]\Big)\|_2^2 + \lambda\|\tilde{u}^{(l)}[k-1]\|_1^2, \quad (5)$$

and (ii) determine $\tilde{B}^{(l)}$ given the values $(\tilde{A}, \{\tilde{u}^{(l)}[k]\}_{k=0}^{N-1})$, which is the solution to the following problem

$$\min_{(\tilde{B}^{(l)})} \sum_{k=1}^{N} \|z[k] - \Big(\tilde{A}z[k-1] + \tilde{B}^{(l)}\tilde{u}^{(l)}[k-1]\Big)\|_2^2 + \lambda\|\tilde{B}^{(l)}\|_1^2. \quad (6)$$

It is important to notice that several criteria can be adopted to stop the sequential procedure in the algorithm. In particular, we have considered the total variation between iterations on the inputs to be below a specified threshold. Lastly, to control the inputs' spatial and temporal sparsity independently for the analysis in S13 Fig and S14 Fig, we used two separate λ values, namely, λ_U and λ_B in steps (5), and (6).

SI2. LTI system's output simulation

We modeled the activity of four hypothetical *observed* regions as a four-dimensional LTI system and the influence of the unobserved regions and external stimuli into each node as an unknown driver. We used MATLAB's *place.m* function to design a synthetic system matrix with eigenmodes oscillating at 0.01 and 0.06 Hz to mimic the frequencies of the BOLD signal's neurophysiological component. We generated 100 different sets of 30-min simulated time series with the HCP dataset's fMRI sampling rate (1.4 Hz) from the same synthetic system matrix. We created external input patterns to mimic the conventional event-related and block-design paradigms (see manuscript Fig 1B). Specifically, we created periods of external stimulation with different durations and frequencies as follows: At period II (9–12 min) of the manuscript Fig 1B, the blue node is stimulated in 25 TRs = 18 second blocks, interleaved with similarly sized rest periods, and at period III (15–18 min) the blue node is stimulated for 7 TRs = 5.04 seconds, with inter-stimulus intervals of 3 TRs = 2.16 seconds. We generate different random normalized time series as the recording noise for each node. We select the external input to internal noise variance ratio = 1/16 and the signal to recording noise = 1000. We select low noise conditions in the manuscript Fig 1 to aid the visualization of the results. Therefore, we explore the effect of different internal and recording noise levels on our ability to extract the external inputs accurately more in-depth in S1 Fig.

Specifically, instead of the aforementioned input patterns, we used two different sample input patterns from the HCP task protocols: the binarized social task regressor in S5A Fig and the visual cue condition of the motor task in S5E Fig. These results show that the measured outputs' magnitude and the statistical effect size are dependent on the input patterns. For instance, the t-values are almost twice as large in panel A than B under similar noise levels. We can also calculate these metrics from the HCP task data. In theory, the comparison between observed and modeled stimulus-induced BOLD changes should offer insight into the extent of internal and recording noise levels. In the S1C Fig, we provide the maximum (across all ROIs and 0-12 TR lags) the normalized magnitude of the task-related changes in the BOLD and their corresponding t-values for six HCP task scans. These results show that the modeled outcomes from the two sample tasks do not align well. We believe this discrepancy can be partly explained by the unaccounted non-linearity (e.g., hemodynamic filtering) and the differences between the empirical and synthetic systems' spectral profile, size, and input foci. Nevertheless, this framework provides a guideline for what to

expect in low or high noise conditions. For instance, these results show that relatively higher levels of external input and high signal-to-noise conditions result in more considerable divergence of the estimated system matrices from the true underlying system, and consequently, a higher estimation error in inputs.

However, as discussed earlier, the exact contributions of internal and external noise and external inputs to the BOLD signal are unknown. Therefore, we only sampled a few noise levels to understand how different noise levels will affect the accuracy of the estimated inputs. Nevertheless, these results show that the signal-to-noise levels close to the possible empirical values can result in different outcomes depending on the pattern of inputs, the utilized system matrix, and the internal noise levels. More importantly, these results provide evidence that the system matrices estimated during periods without stimulation are only beneficial in relatively higher input magnitude and signal-to-noise levels. Together, these observations combined with the main manuscript results demonstrating the utility of system matrices estimated during resting-state in the accurate identification of external inputs, suggest the relatively high levels of external input's magnitude during task scans.

SI3. The dimensionality of external inputs.

One of the most critical parameters of the model is the input matrix B dimensions. This matrix gives us information on the inputs' spatial patterns. However, the dimensions of these external inputs are not known a priori, and naturally, our ability to accurately capture the external inputs depends on this critical parameter. Specifically, input matrices B with dimensions lower than those of the external inputs will not extract all input patterns correctly, whereas higher dimensional input matrices are likely to capture some of the internal and recording noise as external inputs.

Therefore, since we do not know the dimensionality of the external inputs in BOLD, we approximated the dimensions of the input matrix B by performing principal component analysis on the residuals of the models. As seen in S19 Fig, principal components 1–25 capture more than 80% of variance in the average residuals and more than average 60% of subject-level residuals' variance across all tasks. In addition, we compared the goodness-of-fit of the LTI model with and without external inputs using Akaike information criterion (AIC)[4]. Our results demonstrate that incorporating external inputs does not lead to overfitting and improves the model's fit – an effect

most pronounced in higher dimensional input matrices (S20 Fig). Finally, we demonstrate that we identify the external inputs during the motor task similarly at high-dimensional input matrices (S18 Fig), as indicated by the high correlation (>0.8) of inputs estimated using input matrix dimensions higher than 25 (S18I Fig).

Together, these results show that higher-dimensional input matrices are required to capture the spatiotemporal profiles of the inputs during the motor paradigm. We expect these results as the motor task conditions activate different specialized brain regions along the somatomotor cortices. Therefore, we select $p = 25$ for input matrix B for the main manuscript results, much higher than the anticipated task conditions yet small enough to minimize the unwanted noise captured by high dimensional inputs. Nevertheless, these additional analyses also demonstrate the robustness of the captured spatiotemporal input profiles to the choice of high dimensional B matrices.

SI4. Determining the optimal number of eigenvector clusters.

To find the optimal number of eigenvector clusters, we used the following methods.

Elbow method

K-means clustering identifies clusters such that it minimizes the within-cluster variance (or the within-cluster sum of squared errors (SSE)). Therefore, ideally, we want this error to be as small as possible. However, increasing the number of clusters always results in a smaller sum of squared errors. The Elbow method is based on plotting SSE as a function of number clusters and identifying a k such that adding another cluster does not result in notable change or reduction in SSE. Generally, the *'elbow'* (bend) in the SSE curve indicates the optimal number of clusters. However, it is worth noting that the elbow is not always readily identifiable, especially if the data is not very clustered.

Calinski-Harabasz criterion

We used the criterion proposed by[5], also known as the variance ratio criterion defined as:

$$VRC = \frac{SS_B}{SS_W} \times \frac{(N-k)}{(k-1)}, \tag{7}$$

where k is the number of clusters, N is the number of observations, and SS_B and SS_W are the total between-cluster and within-cluster variance, respectively. SS_B and SS_W are defined as:

$$SS_B = \sum_{1}^{k} n_i \left\| m_i - m \right\|^2, \tag{8}$$

$$SS_W = \sum_{1}^{k} \sum_{x \in c_i} \left\| x - m_i \right\|^2, \tag{9}$$

where k is the number of clusters, x represents a data point, c_i represents the i_{th} cluster, n_i is the number of points in the i_{th} cluster with the centroid

m_i, m is the total mean of the data, $\|x - m_i\|$ and $\|x - m_i\|$ the Euclidean distance (L^2 norm) between the two vectors. Since well-defined clusters display a high between cluster variance and low within-cluster variance, we find the optimal number of clusters by identifying a k that maximizes the variance ratio criterion.

Davies-Bouldin criterion

The Davies-Bouldin criterion[6] captures the ratio of within- and between-cluster distances and is defined as

$$DB = \frac{1}{k} \sum_{i=1}^{k} \max_{j \neq i} D_{i,j}, \tag{10}$$

where $D_{i,j}$ is the ratio of within-to-between cluster distance for clusters i and j, which is defined as

$$D_{i,j} = \frac{(\bar{d}_i - \bar{d}_j)}{d_{i,j}}, \tag{11}$$

where $\bar{d}_i$ and $\bar{d}_j$ are the average distances between each data point in the i_{th} and j_{th} clusters to their own cluster centroids. $d_{i,j}$ is the distance (Euclidean) between the centroids of clusters i and j. Therefore, the optimal k minimizes the Davies-Bouldin index and represents the best within-to-between cluster distance ratio.

Silhouette criterion

Silhouette criterion[7] is a measure of similarity of each point to other points in its cluster, compared to data points in other clusters and is defined as:

$$S_i = \frac{a_i - b_i}{\max(a_i, b_i)}, \tag{12}$$

where a_i is the average distance between the i_{th} data point to the other points in its own cluster, and b_i is the minimum average distance between i_{th} data point to data points in different clusters (minimized across all clusters). Therefore, a high Silhouette score (ranging between 1 and -1) indicates that the data point is well grouped within its own cluster and poorly matches

the data points from other clusters. Conversely, many data points with zero or negative Silhouette values indicate the presence of few or many clusters in the data.

Prior research has demonstrated inhomogeneity in the spectral profile of the BOLD signal across the brain[8;9;10;11]. Hence, we hypothesize that there is inhomogeneity in the frequency and damping of eigenmodes. To capture the spatial patterns of activity, we use our method to estimate the internal system parameters from the resting-state time series (1200 TR $\approx$ 14.5 min). To identify the eigenmodes with similar spatial patterns across subjects, we aggregate all subjects' eigenvectors and perform k-means clustering analysis.

We show the results of several different criteria for determining the optimal number of clusters in the S3 Fig. These results demonstrate that the optimal number of clusters determined by three different criteria (S3.A-C Fig) is $k = 2$. As seen in S2 Fig, these two eigenvector clusters correspond to the *fast damping rate* eigenmodes, mainly overlapping on the limbic and ventral attention/salience (VN/Sal) canonical resting-state networks (RSNs)[12], and the *slow damping rate* eigenmodes, mainly overlapping on dorsal attention (DN) and visual (Vis) networks. It is also worth noting that the sensory/motor (SM), followed by executive control (ECN) and default mode network (DMN), overlap across both groups.

However, S3D Fig demonstrates that $k \approx 4$ roughly corresponds to the elbow of the sum of the squared error curve (as a function of the number of clusters). Manuscript Fig 3 demonstrates that $k = 4$ further divides the stable and unstable clusters into smaller spatial clusters with significantly (bootstrap $n = 50,000$, Bonferroni corrected $p < 0.05$) different frequency and stability profiles (except for the frequency of clusters 3 and 4). Together, these results highlight the spatial heterogeneity and diversity of the oscillatory mode's frequency and stability profiles.

SI5. The sparsity of system matrix and external inputs.

The high dimensionality of the BOLD signal poses a problem from the model fitting perspective as the highly parameterized models can overfit. Therefore, a good fit in such models may not indicate an accurate identification of the underlying parameters, but instead, the overabundance of parameters can lead to misidentified parameters and small residuals. Thus, it is beneficial to test the sensitivity of the estimated system and inputs' parameters and the model's goodness-of-fit. Therefore, in addition to the least-squares method detailed in SI4, we estimated sparse system matrices from the resting-state time series using LASSO regularization. We estimated the sparse system matrices using 0.1, 0.01, and 0.001 regularization parameters that result in roughly 95%, 50%, and 5% sparsity. Commonly to account for overfitting, a cost function is defined comprising the error and the number of parameters to penalize over-parameterization. We used Akaike information criterion (AIC)[4] to test the effect of system matrix sparsity (i.e., number of non-zero parameters) on the model's goodness-of-fit. Following[13], we used $AIC = m \ln(RSS/m) + 2k$ to calculate AIC values (m number of observations, k number of parameters, and RSS residual sum of squares). S12 Fig shows the AIC values for A matrices with different sparsity levels and different input matrix B dimensions. These results suggest that full system matrices in the main manuscript are not likely overfitting the data, as indicated by notably higher AIC values of sparse matrices.

Next, we examined the effect of input sparsity on the input estimation accuracy using a multiple linear regression model and the known task regressors. In the main text, we tuned both input U and input matrix B with a single regularization parameter, which resulted in $6.76 \pm 0.8\%$ and $5.91 \pm 0.4\%$ average zero elements in B and U, respectively. Here, in order to control the sparsity of U and B independently, we introduced two separate spatial and temporal regularization factors, namely λ_B and λ_U, respectively. Sweeping both regularization parameters allows us to identify subject-specific λ_B and λ_U values that result in 20%, 50%, and 80% sparsity in both U and B matrices (S13A-B Fig). Overall our results suggest that increasing the sparsity of inputs beyond 20 % results in reduced accuracy of the estimated inputs (S13C Fig).

We also performed PCA on the estimated inputs concatenated across all subjects and identified the subject-level input with the highest loading for PCs 1–10. Finally, we repeated this analysis for different input sparsity

and input matrix B dimensions. S14A Fig shows the mean accuracy of the estimated inputs. Since the same input structures can manifest in different PCs depending on the input sparsity and input matrix B dimensions, we performed k-means ($k = 10$) clustering on the spatial profiles of the inputs (S14B Fig). We identified these spatial patterns from the $t-$test values of brain regions with significant ($t-$test, $p < 0.05$, FDR corrected for multiple comparisons across ROIs) loadings on input matrix B rows corresponding to the aforementioned PCs. S14C Fig shows the centroid of the identified clusters 2–7. We did not present cluster 1 since all the values were zeros. Together these results suggest that although overall high input sparsity can reduce the estimation accuracy, the input sparsity and dimensions can be further tuned to capture different input patterns optimally.

SI6. Effect of estimation window's length on input's accuracy.

We leveraged the full-length ($\approx$ 14.5 min) resting-state time series to estimate the system parameters due to the high dimensions of the system and the slow oscillations in the BOLD. Here we examined the effect of window size on the accuracy of the estimated inputs. We provide the mean accuracy of the subject- and group-level (mean) estimated inputs for all brain regions across several lags for a sample task (Motor). These results show that the subject-level system matrices estimated using both short (3–minute) and full-length resting-state time series increase the estimated inputs' accuracy (measured via R^2 of the multiple linear regression fit) compared to system matrices estimated during the task scan (S8 Fig). Although we find similar results are the group-level average means, interestingly, the system matrix estimated using short window results in overall higher input accuracy. For instance, the R^2 values are higher in the early visual ROIs for full-length window estimates, but the vast majority of the inputs other ROIs have higher R^2 values in the system estimated using the short window.

In addition, PCA analysis on the estimated inputs similar to the manuscript Fig 6 reveals that analogous components emerge in both short and full-length window estimates. As seen in S15 Fig, PC 1–4 are identical on panels C and D. Nevertheless, statistical compassion between the inputs corresponding to each PC reveals that the estimated inputs' time series are significantly (Wilcoxon rank-sum test, $p < 0.05$) more accurate (measured via R^2) for the aforementioned PCs in short window estimates. We speculate that the resting-state scan's unaccounted inputs likely contribute to the higher error in the full-length estimation window. In addition, the inhomogeneous distributions of eigenmodes' spatiotemporal profiles can also play a role, as longer estimation windows can lead to inaccurate estimation of higher frequency and transient activity, especially in the presence of high-frequency noise. Together, these results show that resting-state estimated system matrices offer a suitable approximation of the underlying system and that our reported findings in the main manuscript are robust to estimation window length.

SI7. Principal component analysis of LTI model's residuals.

In addition to our proposed method for estimating external inputs based on spatial and temporal regularization of the LTI model's residuals, we also decomposed residuals using the principal component analysis to investigate the robustness of the estimated inputs' profiles to the choice of factorization method. We concatenated residuals' PCs 1–25 across all subjects and performed a group-level principal component analysis. We provide the temporal profiles of the group-level residuals' PCs 1–25 in S16A Fig. We find that both methods similarly identify the aforementioned group-level estimated input PCs 1–7, as indicated by high absolute correlation values between the corresponding estimated input PCs and residual PCs (S16B–C Fig). However, estimated input PCs 8 and 9 (S11 Fig) are the ones most similar to the residual PC 8 (S16B Fig). Closer examination of temporal profiles of estimated input PCs 8 and 9 (S11 Fig), as well as several of the remaining estimated input PCs (S16A Fig), show that as mentioned earlier, these estimated input PCs capture a rapid sequence of inputs to different brain regions following the task block's offset. S8A Fig (bottom panel) shows that these rapid changes following the task block's offset are not present in the group-level residual PCs. These results suggest that despite the overall high similarity between characterized spatiotemporal profiles of external inputs across both methods, the spatiotemporal regularization approach is more likely to identify the spatially sparse and transient inputs.

Supplementary Figures

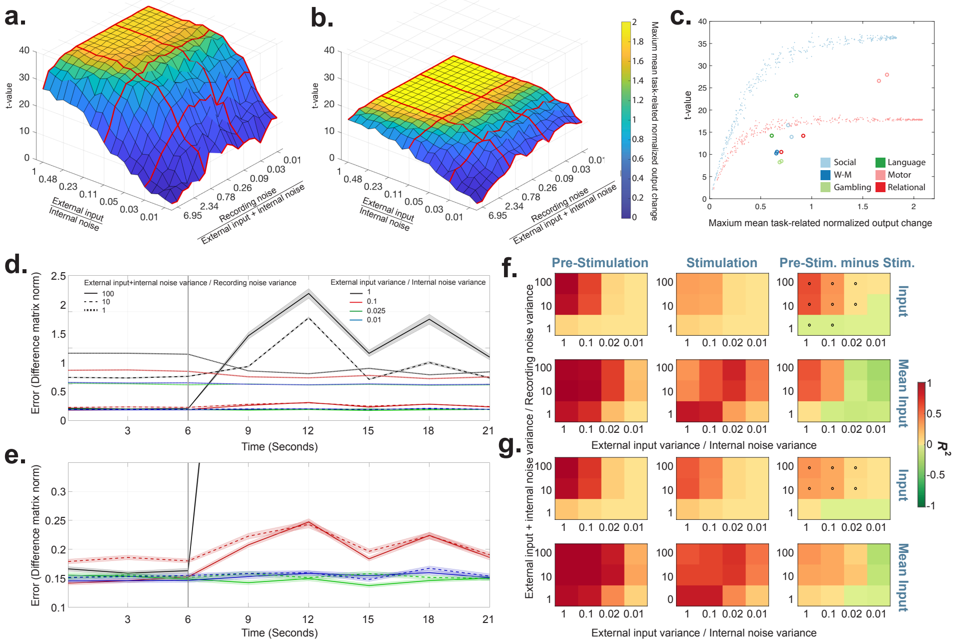

Figure 1: **The simulated input-induced dynamics with different internal and recording noise levels.** ***(A-B)*** The color-coded surface shows the average magnitude and variance of the input-induced changes in the system outputs. Specifically, the colors indicate the maximum (across all regions) average magnitude of the input-related changes in the normalized outputs, and the z-axis shows the t-values calculated from comparing the simulated outputs between periods with and without inputs. The x-axis shows the internal noise to external input ratio, and the y-axis shows the total drivers (i.e., external input plus internal noise) to the recording noise ratio. We used the same synthetic four-dimensional system in manuscript Fig 1, leveraged the binarized version of the patterns in S5A and E Fig as inputs in panels ***A*** and ***B***, respectively. Panel ***C*** shows the same results as panels ***A*** and ***B*** with filled light blue and light red markers. The color-coded open circles show the same results calculated from the HCP task datasets (see Supplementary Information section SI2 for details). ***(D-E)*** The average 2-norm and standard error (100 simulations) of the difference between the system's true and estimated matrices were calculated using a 3–minute sliding window. ***(F-G)*** The estimated inputs' accuracy over two sample time points (12 and 18 seconds time points) was calculated using the R^2 values of the linear fit of the estimated to true inputs. We used system matrices estimated during periods without (left) and with (middle) stimulation. The results on the top row show the average R^2 calculated from estimated inputs at each repetition, and the bottom row, the average input's R^2 values. The red lines in panels ***A*** and ***B*** show the noise levels selected in panel ***F-G*** analyses. The black dots show the noise levels with significantly (Wilcoxon rank-sum, $p < 0.005$) different accuracies (i.e., system matrices estimated from periods with versus without stimulation).

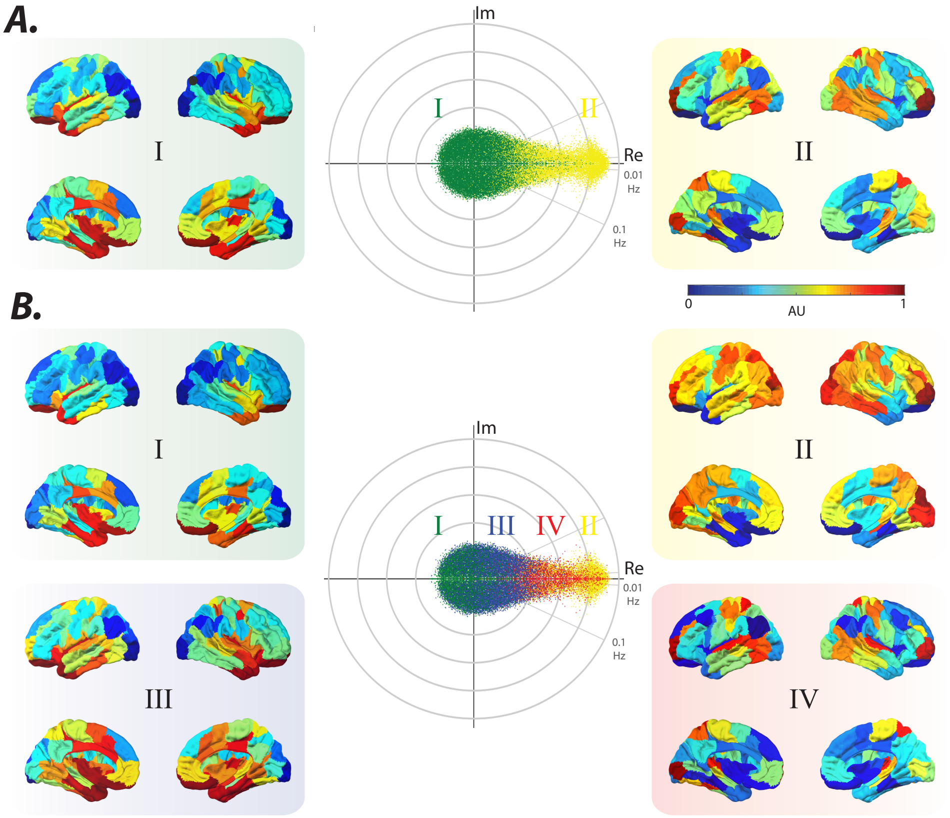

Figure 2: **Low-order eigenvector clusters estimated from the full (1200 TR** $\approx$ **14.5 min) resting-state time series.** Clustering the eigenvalues based on their eigenvector's similarity highlights the spectral profile of different systems. ***(A)*** all eigenvectors from all subjects were normalized and clustered into 2 clusters and ***(B)*** shows 4 clusters using the k-means clustering algorithm with k =4. We color-coded the clusters identified across all subjects and sessions (99 subjects $\times$ 4 sessions $\times$ 100 eigenmodes, i.e., 39600 eigenvalues). The brain overlays represent a spatial distribution of the eigenvector associated with an eigenvalue at the centroid of each cluster (displayed with the same color code). To aid the visualization, the centroids were normalized after subtracting from each centroid its minimum element. The color-bar represents the normalized values of centroids for all clusters.

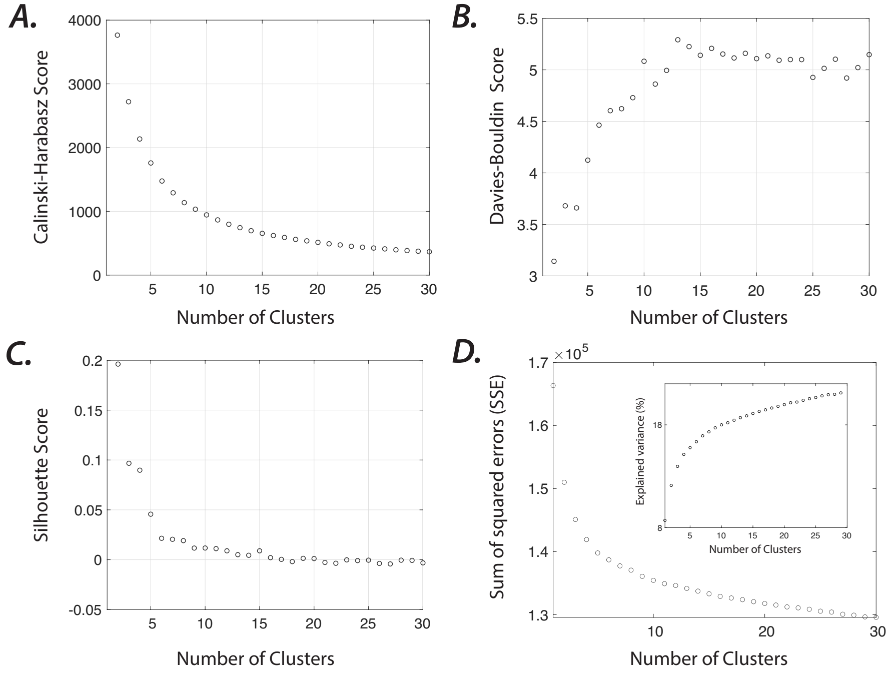

Figure 3: **Determining the number of eigenvector clusters.** To evaluate the optimal number of clusters (k) present in the eigenvectors we used ***(A)*** Calinski-Harabasz criterion (i.e., the ratio of within to between cluster dispersion)[5], ***(B)*** Davies-Bouldin criterion (i.e., the ratio of within-cluster and between-cluster distances)[6], and ***(C)*** Silhouette criterion (i.e., the measure of how similar each data point is to other points in its cluster, compared to points in other clusters)[7]. Based on all three criteria, $k = 2$ is optimal number clusters in the data – see SI4 for more details regarding these criteria. ***(D)*** The plot represents the sum of squared errors (i.e., distances to cluster centers), and the inset plot the percent variance explained by the different number of clusters. Note that due to the smoothness of the curve, we can not identify a single k that corresponds to the *'elbow'* of the curve in panel ***D***. Together, these results indicate that the data may not contain clearly defined clusters at $k > 2$. Therefore to examine the organization of eigenvectors at higher resolutions, in the main manuscript, we choose $k = 4$ that roughly corresponds to the curve's elbow.

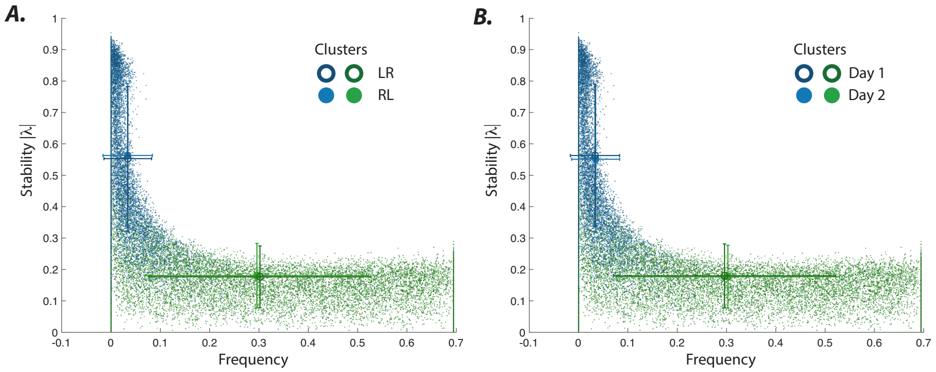

Figure 4: **Comparison of eigenvalues' frequency and stability across different image acquisition types and sessions.** ***(A)*** Frequency and stability of eigenvalues separated based on phase-encoding acquisition type. The open and closed circles mark eigenvalues from LR and RL phase-encoding sessions, respectively. The identified k-means eigenvector clusters (k=2) are color-coded. The error bars indicate the mean and standard deviation of clusters? stability and frequency. ***(B)*** Frequency and stability of eigenvalues separated based on acquisition session. The open and closed circles mark eigenvalues from days 1 and 2. Statistical comparisons (bootstrapping n=50,000) failed to show any difference between the distribution's frequency and stability in both panels ***A*** and ***B***.

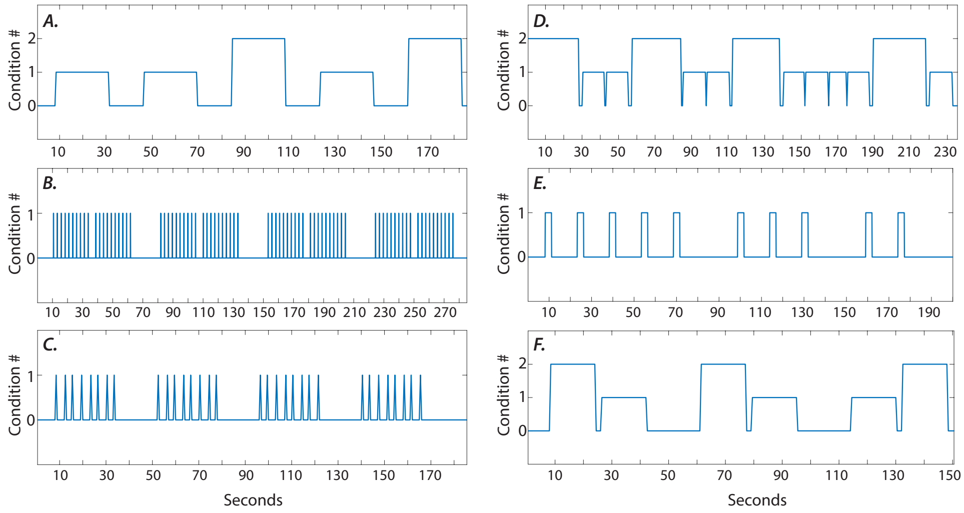

Figure 5: **Sample session's task regressors *(A–F)*** A sample scanning session's boxcar regressor for different task conditions (social, working memory, gambling, language, visual cue condition of motor, and relational tasks, respectively). Different task cues and/or blocks are marked by '1' and '2' on y-axis, whereas '0' represents inter-stimuli rest intervals. Since the spectral profile of the task regressors are identical across subjects, we used regressors in **(A–F)** for our analysis.

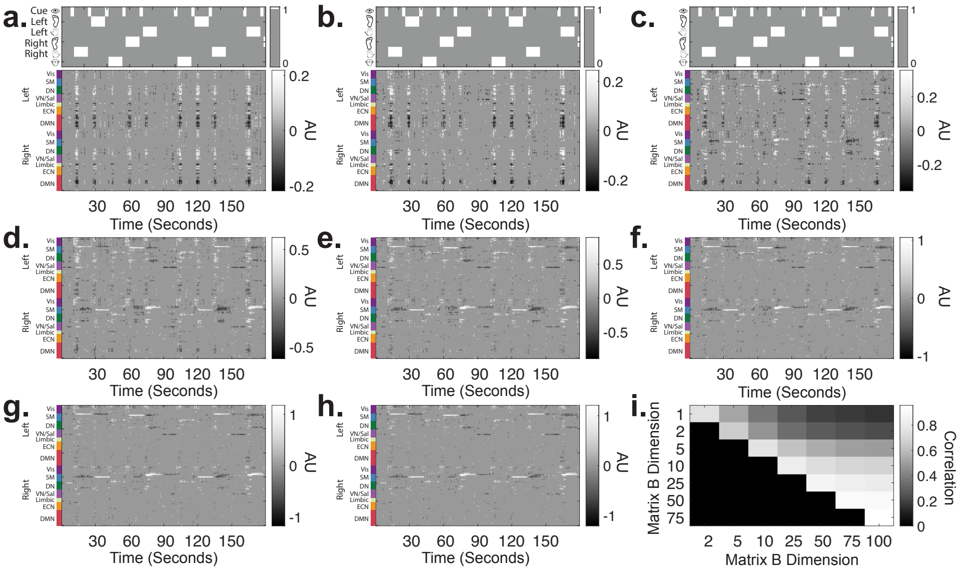

Figure 6: **Average estimated inputs and input matrix B dimension.** Internal system parameters (i.e., A matrices) during full-length resting-state and motor scans were used to estimate the external inputs ($B \times U$) in panels ***A-H***. We used input matrix B with 1, 2, 5, 10, 25, 50, 75, and 100 in panels ***A-H***, respectively. Time points without significantly higher or lower average inputs estimated during motor task than resting-state scans (paired $t-$test, $p < 0.05$, false discovery rate (FDR) corrected for multiple comparisons) presented in gray (i.e., 0). Top plots in panels ***A-C*** show onsets and durations of visual cues and motor task conditions – left foot, left hand, right foot, right hand, and tongue movement blocks. ***(I)*** Pairwise correlation between all possible pairs of vectorized input matrices in panels ***A-H***.

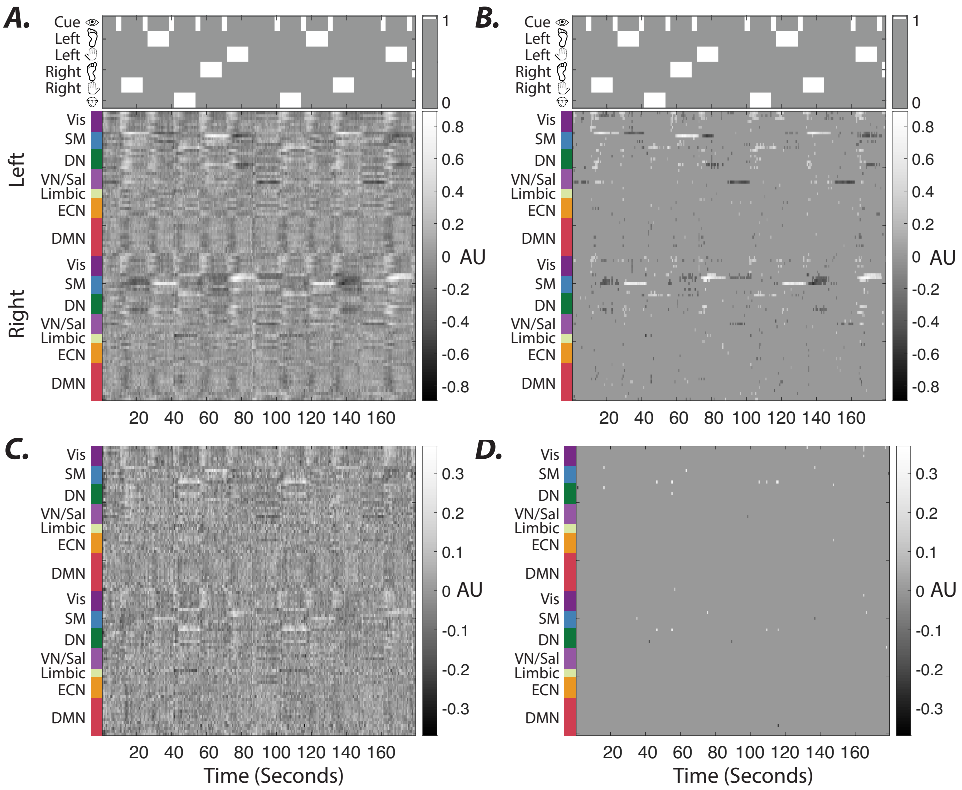

Figure 7: **Average estimated external inputs for the motor task**. Internal system parameters (i.e., A matrices) during full-length resting-state and motor scans were used to estimate the external inputs in panels $\boldsymbol{A}$ and $\boldsymbol{C}$, respectively. Panels $\boldsymbol{B}$ and $\boldsymbol{D}$ show time points form panels $\boldsymbol{A}$ and $\boldsymbol{C}$ with significantly higher or lower average inputs estimated during motor task than phase-randomized null time series (paired $t-$test, $p < 0.05$, false discovery rate (FDR) corrected for multiple comparisons). Top plots in panels $\boldsymbol{A}$&$\boldsymbol{B}$ show onsets and durations of visual cues and motor task conditions – left foot, left hand, right foot, right hand, and tongue movement blocks.

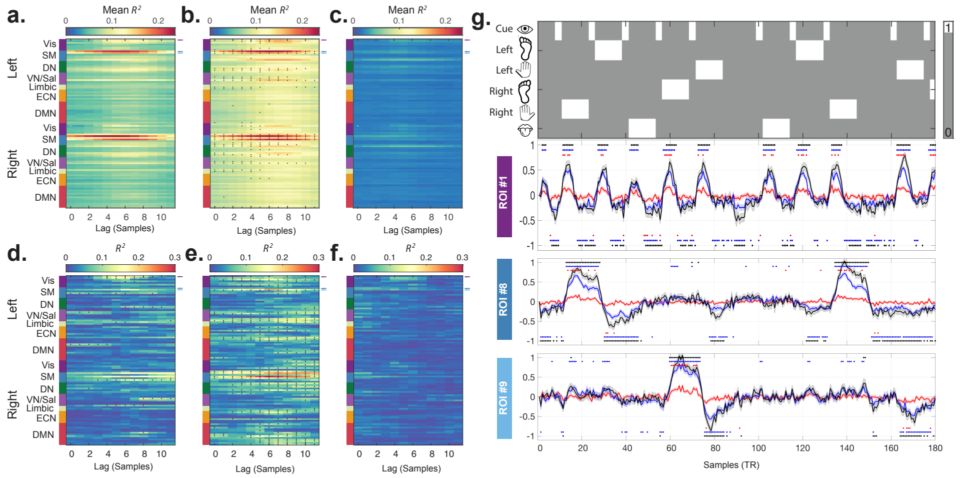

Figure 8: **Goodness-of-fit of multiple linear regression model of estimated external inputs** ***(A)*** The average R^2 values across participants were calculated using multiple linear regression models of estimated external inputs across all brain regions. We also tested various lags (samples) between modeled inputs and the known task (motor) regressors to account for hemodynamic response delays. We used system parameters estimated from the full-length resting-state time series to identify external inputs in panel ***A***. ***(B)*** Same results as panel ***A***, except that we used system parameters estimated from the windowed (250 samples) resting-state time series to identify external inputs. Statistical comparisons (paired $t-$test, $p < 0.05$, Bonferroni corrected for multiple comparisons across all regions and lags) between panels ***A*** and ***B*** reveal that overall R^2 values are similar in brain regions with the highest values (e.g., somatomotor (SM) regions). Nevertheless, R^2 values in other brain regions with lower R^2 values are significantly higher in panel ***B*** Comparisons that did not pass the significance level are marked with black dots (i.e., '.'). ***(C)*** Same results as panel ***A***, except we used system parameters estimated from the motor task time series to identify external inputs. Statistical comparisons (paired $t-$test, $p < 0.05$, Bonferroni corrected for multiple comparisons across all regions and lags) between the results in panels ***A*** and ***C*** reveal that overall the R^2 values are significantly higher in panel ***A***. Comparisons that did not pass the significance level are marked with a white dots. Statistical comparisons (paired $t-$test, $p < 0.05$, Bonferroni corrected for multiple comparisons across all regions and lags) between the results in panels ***B*** and ***C*** reveal that all R^2 values are significantly higher in panel ***B***. ***(D-F)*** Same as in panels ***A-C*** except the result reflect the R^2 values calculated from the group average estimated inputs. The black dots show the R^2 values above one standard deviation across all three panels. ***(G)*** The average (and standard error) estimated inputs to three sample ROIs from the Visual (Purple) and somatomotor (SM) networks (blue). The blue, black, and red lines correspond to results from panels ***D-F***. The three sample ROIs are also indicated by color-coded (based on panel ***G***) arrows in panels ***D-F***.

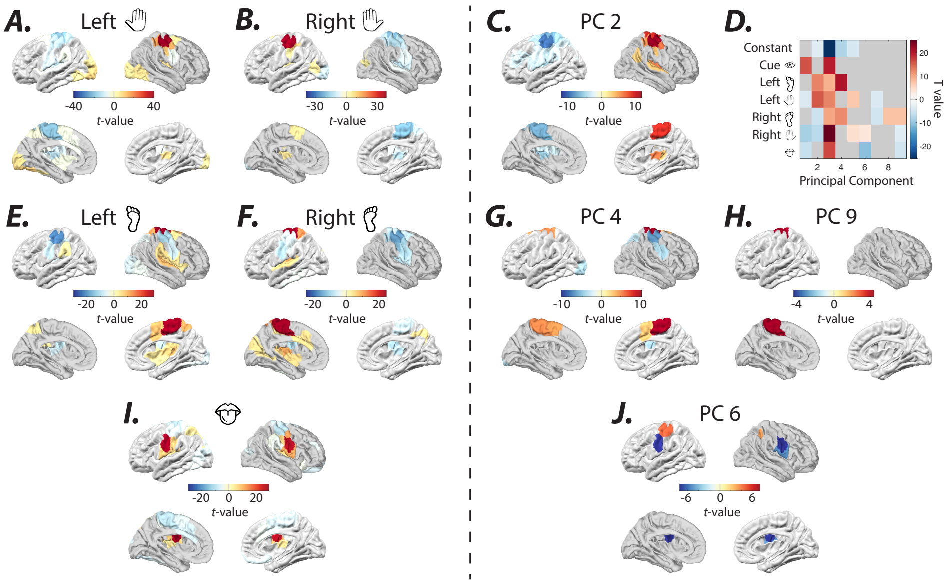

Figure 9: **Motor task activation and their corresponding estimated external inputs.** Panels ***A,B,E,F, and I*** show BOLD activation maps for the left hand, right hand, left foot, right foot, and tongue movement. The color-coded maps indicate $t-$test values of brain regions with significant ($t-$test, $p < 0.05$, Bonferroni corrected for multiple comparisons over regions) coefficients of the general linear model (i.e., task condition versus baseline contrast). For this analysis we convolved task regressors with the hemodynamic response function (HRF) using *spm_hrf.m* MATLAB script from the SPM toolbox[14]. To account for hemodynamic delay variability over brain regions, we created 7 different sets of regressors with HRF function's peaks varying from 3 to 9 seconds (relative to the onset with one second increments). For each region, we fit 7 independent GLMs using these regressor sets and used the GLM coefficients with the highest R^2 values to compute the activation maps. The brain regions identified in these panels match the prior reported task-specific BOLD activations[15;12;16]. Color-coded panels ***(C,G,H,J)*** show the $t-$test values of brain regions with significant ($t-$test, $p < 0.05$, FDR corrected for multiple comparisons across ROIs) loadings on input matrix B rows corresponding to PCs 2, 4, 9, and 6. ***(D)*** $t-$values calculated from coefficients of multiple linear regression models of estimated external inputs associated with each PC (see methods for details). The average coefficients that fail to pass the significance-level across subjects ($t-$test , $p < 0.05$, Bonferroni corrected for multiple comparisons) are depicted in gray.

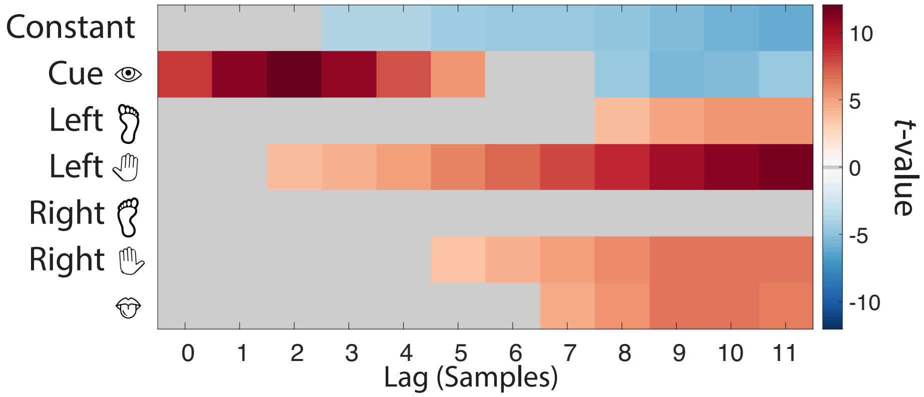

Figure 10: **Multiple linear regression of estimated input's PC 5 using time-shifted regressors.** $t-$values calculated from coefficients of multiple linear regression models of estimated external inputs associated with PC 5 identified in manuscript Fig 6. Since estimated inputs activity seem to lag behind task regressors, we preformed the regression analysis using 12 different sets of time-shifted task regressors (from 0 to 11 samples shift (TR= 0.72 sec)). The coefficients that fail to pass the significance-level across subjects ($t-$test , $p < 0.05$, Bonferroni corrected for multiple comparisons) are depicted in gray.

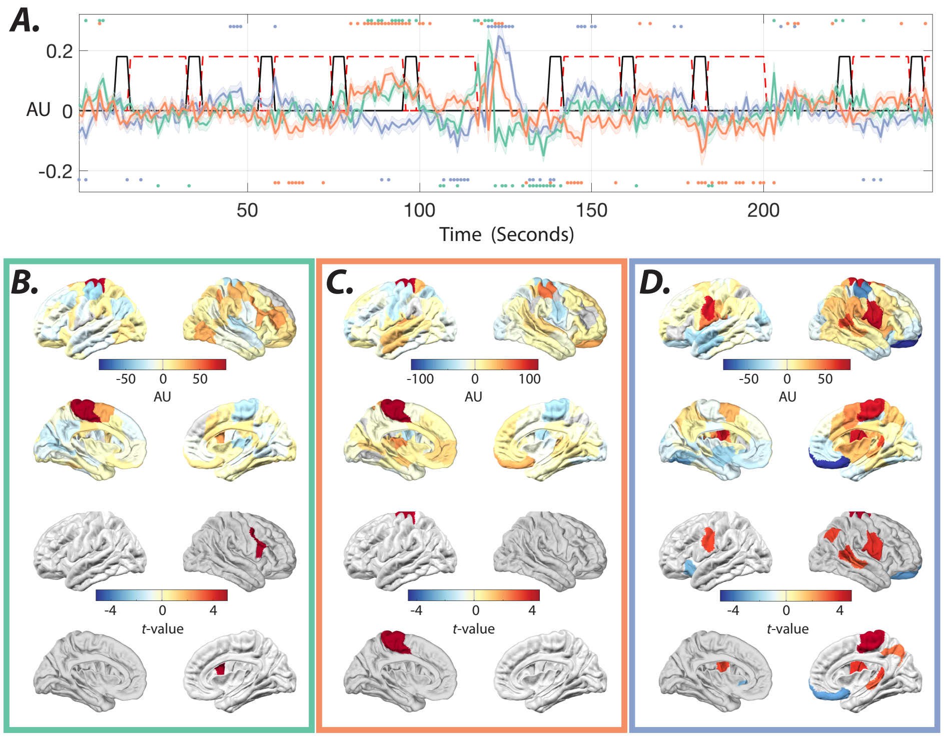

Figure 11: **Temporal and spatial profiles of estimated external inputs associated with the transition from task to baseline.** ***(A)*** Color-coded lines show the mean and standard error (shaded area) of estimated inputs with the highest subject-level loadings for PCs 7 (blue), 8 (green), and 9 (orange). Time points with significant ($t-$test, $p < 0.05$, FDR corrected for multiple comparisons over time) divergence from zero are marked with color-coded dots. The black and dashed red lines show the visual cue and motor task blocks, respectively. Color-coded panels ***B-D*** show the average of input matrix B rows corresponding to the aforementioned PCs (top), and the $t-$test values of brain regions with significant ($t-$test, $p < 0.05$, FDR corrected for multiple comparisons across ROIs) input matrix B loadings (bottom). Note that the average input time series captures the fast sequence of external inputs to brain regions in panels ***B-D*** following the offset task and onset of inter-stimulus interval (i.e., no task fixation baseline).

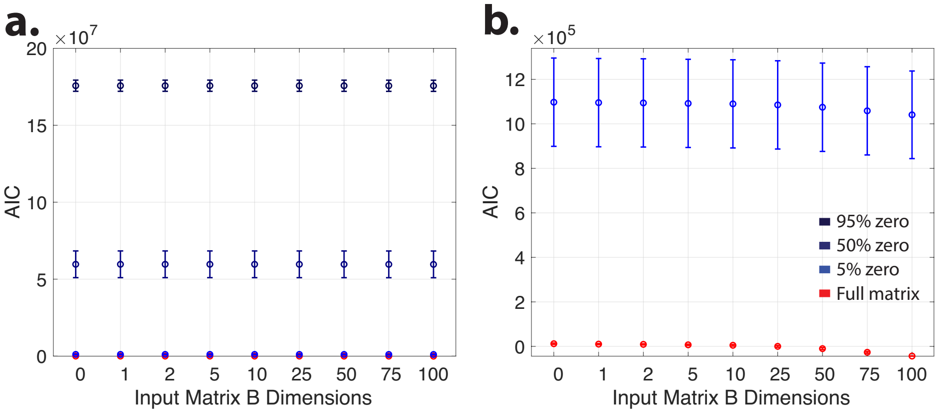

Figure 12: **Sparsity of the system matrix A and model's goodness-of-fit.** ***(A)*** We assessed the model's goodness-of-fit using Akaike information criterion (AIC)[4] for a sample task (motor) and different levels of sparsity constraints on the system matrices. Scatter plots show the mean AIC, and the error bars the standard deviations for different input matrix B dimensions and color-coded sparsity levels. We used system matrices estimated from subjects' full-length resting-state fMRI scans as the system matrices. Following[13], we used $AIC = m\ln(RSS/m) + 2k$ to calculate AIC values (m number of observations, k number of parameters, and RSS residual sum of squares). Statistical comparisons reveal that AIC values are significantly ($t-$test, $p < 0.05$, Bonferroni corrected for multiple comparisons) larger (i.e., worse fit) when sparsity is increased. ***(B)*** same as panel ***A*** zoomed over smaller AIC values.

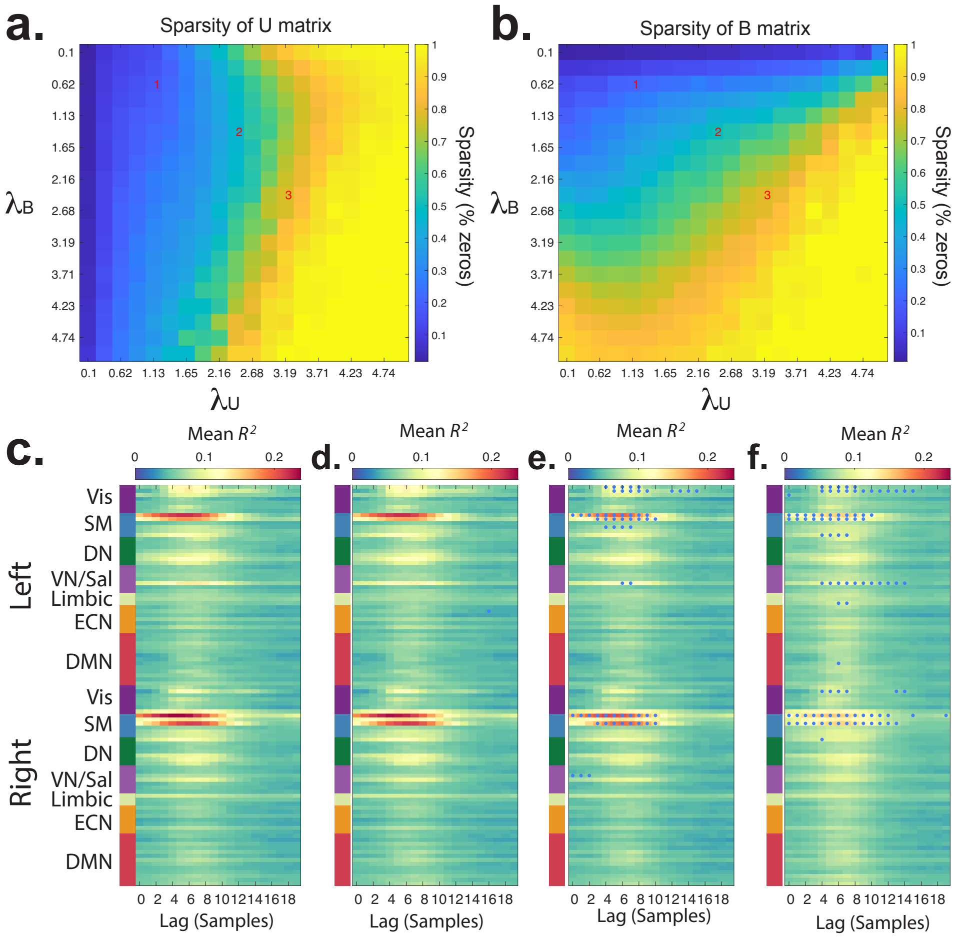

Figure 13: **Estimated input's sparsity and accuracy.** The sparsity values (i.e., percentage of zero elements) of the estimated inputs U ***(A)*** and input matrix B ***(B)*** for a sample subject, calculated over different temporal (λ_U) and spatial (λ_U) regularization factors. Red numbers 1-3 indicate the identified regularization factors that lead to both input matrices displaying 20, 50, and 80 % sparsity, respectively. ***(C)*** The average R^2 values were calculated using multiple linear regression models of estimated external inputs across all brain regions. Inputs are estimated using the regularization parameter used in the main manuscript ($\lambda = 0.5$), which results in low sparsity U ($5.91 \pm 0.4\%$) and B ($5.91 \pm 0.4\%$) matrices. We also tested various lags (samples) between modeled inputs and the known task (motor) regressors to account for hemodynamic response delays. ***(D-F)*** Same results as panel ***C***, but we identified the subject-specific spatial (λ_B) and temporal (λ_U) regularization parameters as detailed in ***(A-B)*** for 20, 50, and 80% input sparsity levels, respectively. Statistical comparisons (paired $t-$test, $p < 0.05$, Bonferroni corrected for multiple comparisons across all regions and lags) between panels ***C*** and ***D*** reveal that overall R^2 values are similar for low sparsity levels. Nevertheless, the mean R^2 values calculated from the inputs to task-related regions (e.g., somatomotor (SM) cortices) are significantly lower in panels ***E&F*** compared to panel ***C***.

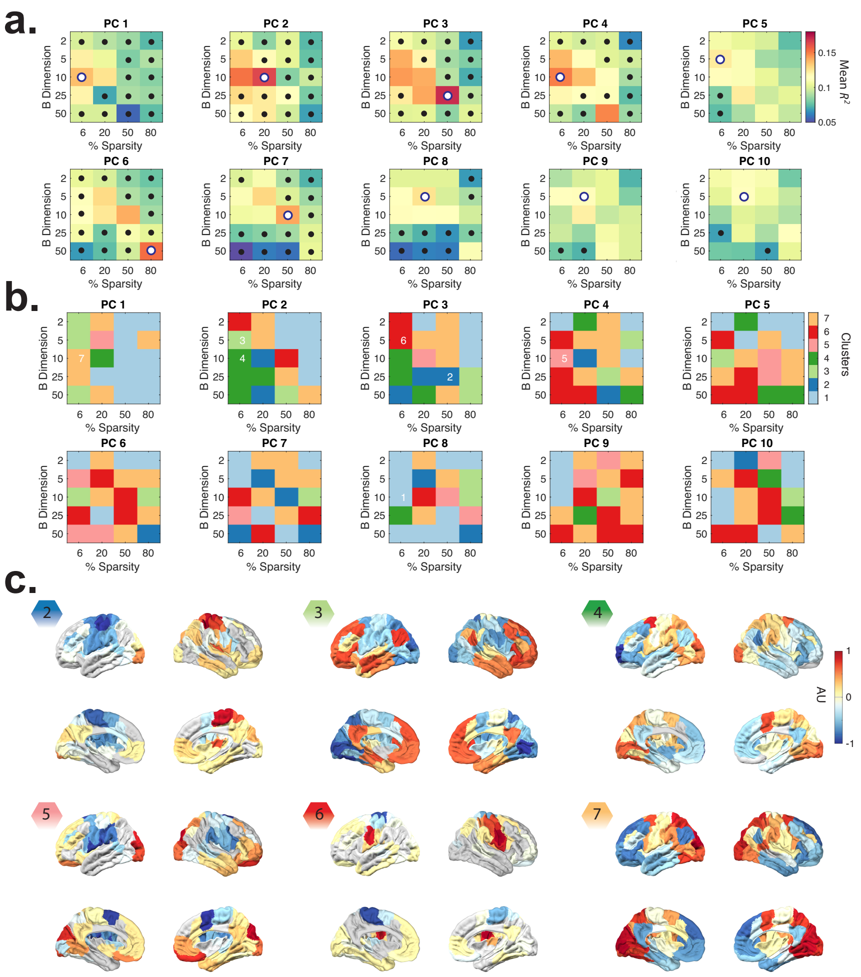

Figure 14: **Input sparsity and estimation accuracy of PC input patterns.** ***(A)*** The average R2 values calculated from subject-level estimated input's PC. For each subject, we only select one component with the highest PC loading for each PCs 1–7. We show the results for different input sparsity (≈6, 20, 50, and 80% zero elements) and input matrix B dimensions (2, 5, 10, 25, and 50). The white dot shows the maximum value for each plot, and the black dots show the elements with significantly lower R^2 (ANOVA, $p < 0.05$, multiple comparisons using multcompare.m script and Tukey-Kramer test). ***(B)*** Next we identify brain regions with significant loading on the estimated input matrix B similar to manuscript Fig 7B-D and performed k-medoids clustering (using absolute correlation values) across all input sparsity and input matrix B dimensions to find similar patterns across different parameter values. Panel ***B*** shows color-coded k-medoids clusters ($k = 7$). The white numbers show the parameters with the highest R^2 values in panel A for clusters 2-7. We show the cluster centroids in panel ***C***, except for cluster 1 with all zero elements (i.e., no significant values across subjects).

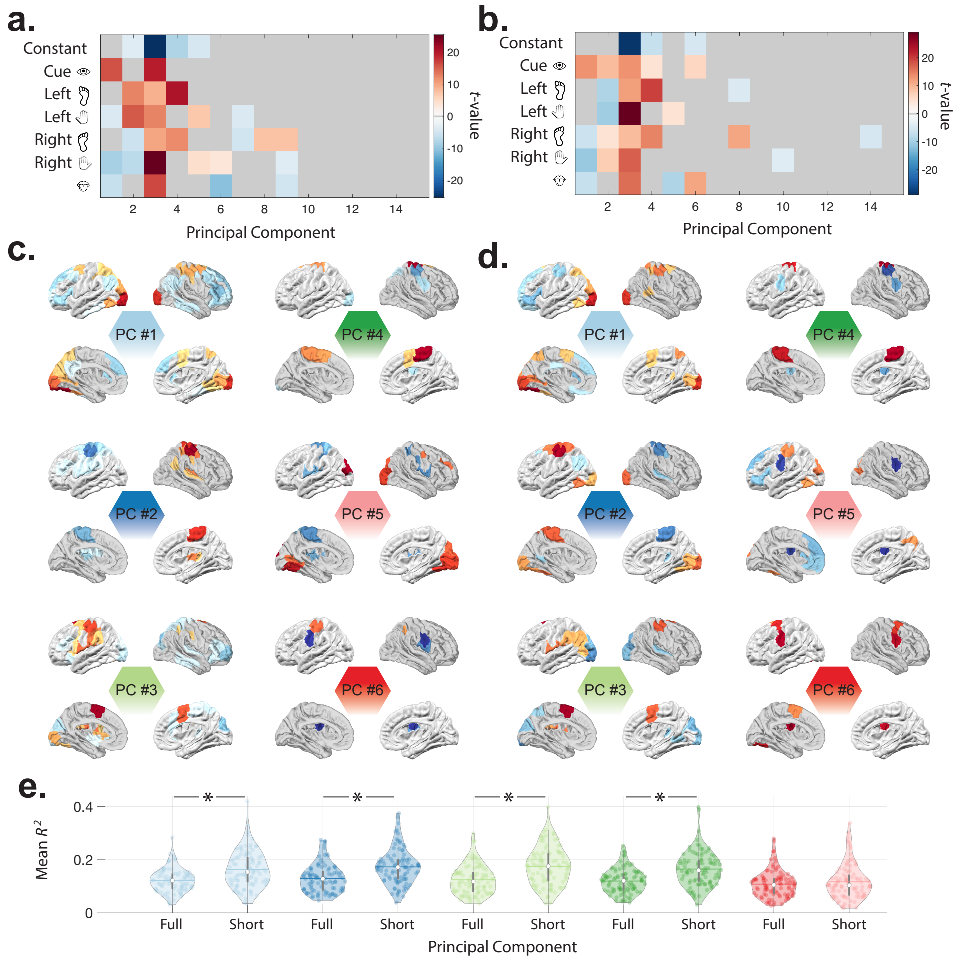

Figure 15: **Principal component analysis of external inputs identified using system matrices estimated from a 3-minute window and full-length resting-state.** ***(A)*** Group-level principal components (PCs) 1–10 calculated from concatenated estimated inputs (input matrix B dimension = 25) across all subjects. ***(A-B)*** t-values calculated from coefficients of multiple linear regression models of estimated external inputs associated with each PC (see methods for details). The average coefficients that fail to pass the significance level across subjects ($t-$test, $p < 0.05$, Bonferroni corrected for multiple comparisons) are depicted in gray. The external inputs in panels A and B are identified using system matrices estimated from a 3-minute window and full-length (≈14.5 minutes) resting-state. Color-coded panels ***(C-D)*** show the $t-$test values of brain regions with significant ($t-$test, $p < 0.05$, FDR corrected for multiple comparisons across ROIs) loadings on input matrix B rows corresponding to the aforementioned PCs1-6. Note that PCs1–4 are similarly identified using both matrices estimated from the full-length ***(C)*** and short 3-minute ***(D)*** windows. ***(E)*** Distributions of R^2 values of multiple linear regression models in panel ***A-B*** for PC1–6, color-coded based on panels ***C-D***. White circles and color-coded horizontal bars indicate the medians and means of distributions, respectively. Pairwise comparison (Wilcoxon rank-sum test, $p < 0.05$, FDR corrected for multiple comparisons) between distributions reveal that R^2 values for principal components marked by '*' are significantly higher in short window system estimates.

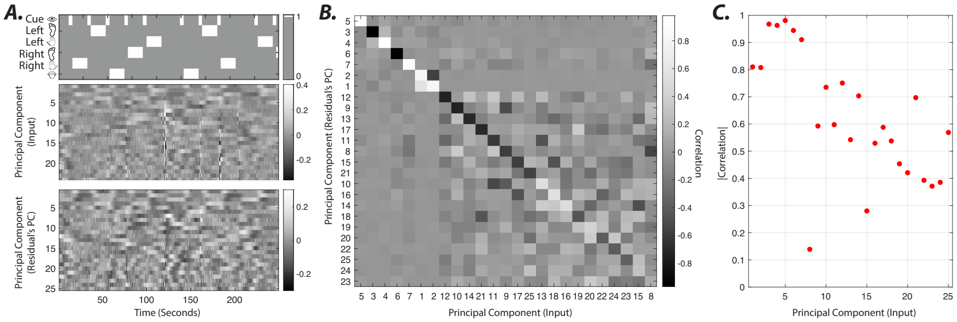

Figure 16: **Factorization of model residuals using spatiotemporal regularization and principal component analysis.** ***(A)*** Top panel show onsets and durations of visual cues and motor task conditions. Middle panel shows the principal components (PCs) 1–15 calculated from concatenated estimated inputs' time series (input matrix B dimension $= 25$) across all subjects. The bottom panel shows PCs 1–15 calculated from the concatenated (across all subjects) first 25 principal components of the LTI model's residuals. ***(B)*** Correlation between estimated inputs and residuals PCs' time series in panel ***A***. Estimated input PCs and residual PCs are sorted by iteratively matching and removing one pair of PCs with the highest absolute correlation. ***(C)*** Correlation values between estimated inputs' PCs 1–25 and their matched residuals' PC time series.

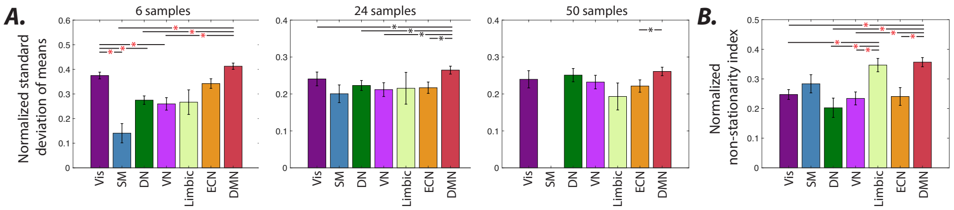

Figure 17: **Non-stationarity of external inputs during resting-state scans *(A)*** The non-stationarity of all brain regions identified in manuscript Fig 8A averaged within each resting-state network. ***(B)*** The non-stationarity of all brain regions identified in manuscript Fig 8B averaged within each resting-state network. The error bars in all panels indicate the standard errors. We used Welch's t-test[17] to test the statistical significance of differences between identified ROIs' estimated inputs' non-stationarity across all possible pairs of resting-state networks. Comparisons that passed the significance-level pre and post FDR correction for multiple comparisons are maker with black and red '*', respectively.

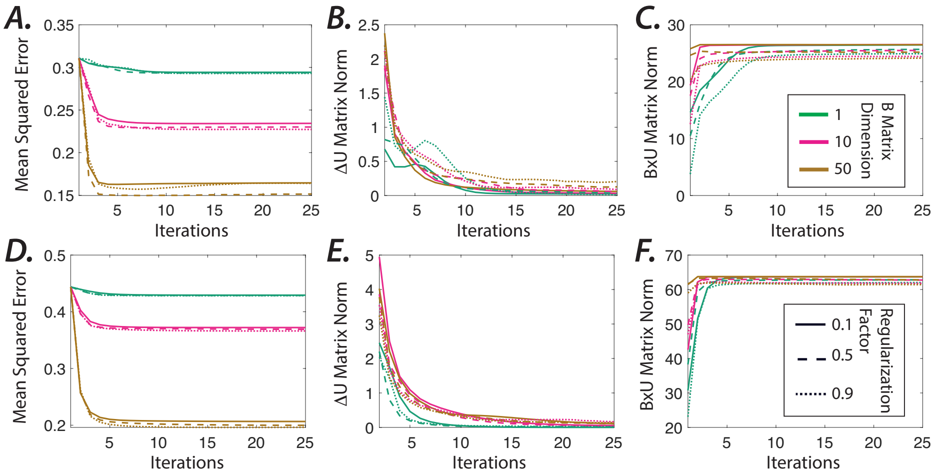

Figure 18: **Convergence of estimated input parameters.** ***(A)*** The mean squared error at different iterations of the estimation algorithm from a sample subject's task (i.e., Social) data. The colors and line styles indicate the dimension of the input matrix and the regularization factors, respectively. ***(B)*** Change in the estimated input matrix (i.e., U) between two consecutive iterations. ***(C)*** The 2-norm of the estimated input (i.e., $B \times U$ matrix). The bottom row panels ***(D-F)*** show the same analyses results estimated from the same subject's full-length resting-state data.

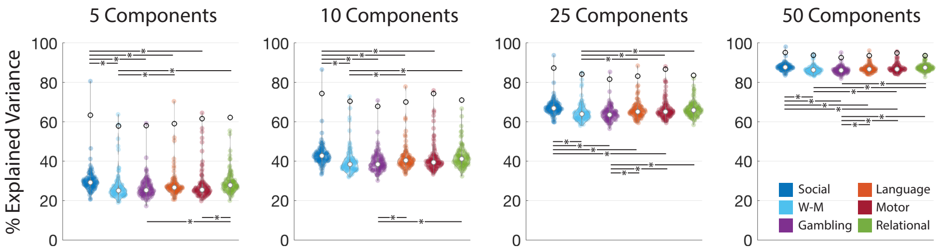

Figure 19: **Percent variance of LTI model's residuals explained by principal components.** Distributions show the sum of the explained variance of the first 5, 10, 25, and 50 principal components of the LTI model's residuals (without input) for six different task conditions. Pairwise comparisons indicate that the same number of components explain significantly (Wilcoxon rank-sum, $p < 0.05$, FDR corrected for multiple comparisons, marked by '*') different percentage of variance across several tasks.

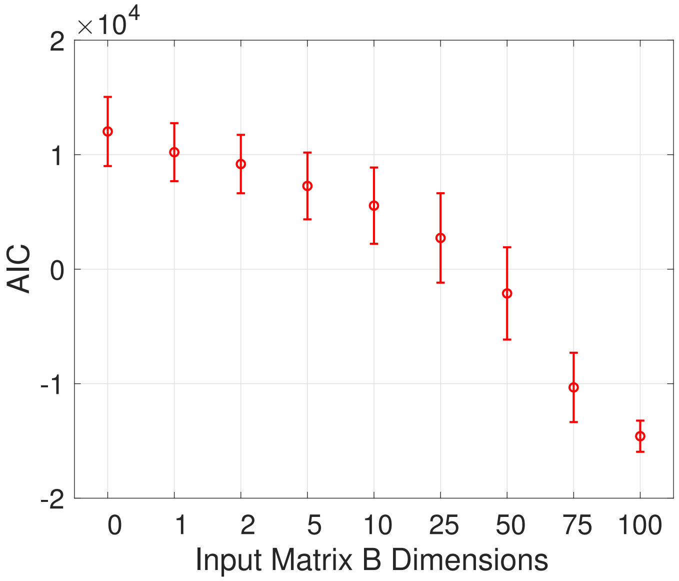

Figure 20: **Goodness-of-fit of LTI model using Akaike information criterion (AIC).** We assessed the model's goodness-of-fit using Akaike information criterion (AIC)[4] for a sample task (motor) with (i.e., input matrix dimension =0) and without external inputs. We used system matrices estimated from subjects' full-length resting-state fMRI scans as the system matrices. Following[13], we used $AIC = m \ln(RSS/m) + 2k$ to calculate AIC values (m number of observations, k number of parameters, and RSS residual sum of squares). Statistical comparisons reveal that AIC values are significantly ($t-$test, $p < 0.05$, Bonferroni corrected for multiple comparisons) smaller (i.e., better fit) when input matrices are included in the model and that higher-dimensional inputs further improve the fit. These results also suggest that adding external input parameters does not lead to over-fitting.

References

[1] Dempster AP, Laird NM, Rubin DB. Maximum likelihood from incomplete data via the EM algorithm. Journal of the Royal Statistical Society: Series B (Methodological). 1977;39(1):1–22.

[2] Gupta G, Pequito S, Bogdan P. Learning Latent Fractional dynamics with Unknown Unknowns. In: Proceedings of the 2019 American Control Conference; 2019. p. 217–222.

[3] Gupta G, Pequito S, Bogdan P. Dealing with Unknown Unknowns: Identification and Selection of Minimal Sensing for Fractional Dynamics with Unknown Inputs. In: Proceedings of the 2018 American Control Conference; 2018. p. 2814–2820.

[4] Akaike H. Information theory and an extension of the maximum likelihood principle. In: Selected papers of hirotugu akaike. Springer; 1998. p. 199–213.

[5] Caliński T, Harabasz J. Communications in Statistics - Theory and Methods. Communications in Statistics. 1974;3(1):1–27. doi:10.1080/03610927408827101.

[6] Davies DL, Bouldin DW. A Cluster Separation Measure. IEEE Transactions on Pattern Analysis and Machine Intelligence. 1979;PAMI-1(2):224–227. doi:10.1109/TPAMI.1979.4766909.

[7] Rousseeuw PJ. Silhouettes: A graphical aid to the interpretation and validation of cluster analysis. Journal of Computational and Applied Mathematics. 1987;20(C):53–65. doi:10.1016/0377-0427(87)90125-7.

[8] Chen JE, Glover GH. BOLD fractional contribution to resting-state functional connectivity above 0.1 Hz. Neuroimage. 2015;107:207–218.

[9] Gohel SR, Biswal BB. Functional integration between brain regions at rest occurs in multiple-frequency bands. Brain connectivity. 2015;5(1):23–34.

[10] Boubela RN, Kalcher K, Huf W, Kronnerwetter C, Filzmoser P, Moser E. Beyond noise: using temporal ICA to extract meaningful information from high-frequency fMRI signal fluctuations during rest. Frontiers in human neuroscience. 2013;7:168.

[11] Niazy RK, Xie J, Miller K, Beckmann CF, Smith SM. Spectral characteristics of resting state networks. In: Progress in brain research. vol. 193. Elsevier; 2011. p. 259–276.

[12] Thomas Yeo B, Krienen FM, Sepulcre J, Sabuncu MR, Lashkari D, Hollinshead M, et al. The organization of the human cerebral cortex estimated by intrinsic functional connectivity. Journal of neurophysiology. 2011;106(3):1125–1165.

[13] Burnham KP, Anderson DR. A practical information-theoretic approach. Model selection and multimodel inference, 2nd ed Springer, New York. 2002;2.

[14] Friston KJ, Zarahn E, Josephs O, Henson RN, Dale AM. Stochastic designs in event-related fMRI. Neuroimage. 1999;10(5):607–619.

[15] Barch DM, Burgess GC, Harms MP, Petersen SE, Schlaggar BL, Corbetta M, et al. Function in the human connectome: task-fMRI and individual differences in behavior. Neuroimage. 2013;80:169–189.

[16] Casorso J, Kong X, Chi W, Van De Ville D, Yeo BT, Liegeois R. Dynamic mode decomposition of resting-state and task fMRI. Neuroimage. 2019;194:42–54.

[17] Welch BL. The generalization ofstudent's' problem when several different population variances are involved. Biometrika. 1947;34(1/2):28–35.
